# To wear or not to wear a mask in the COVID-19 era? The broken bridge between recommendations and implementation in Lebanon

**DOI:** 10.7189/jogh.10.020311

**Published:** 2020-12

**Authors:** Tamar Kabakian-Khasholian, Jihad Makhoul, Marco Bardus

**Affiliations:** Department of Health Promotion and Community Health, Faculty of Health Sciences, American University of Beirut, Lebanon

At the beginning of the COVID-19 pandemic, the scientific community has been debating the use of face masks amongst the general public, sending conflicting recommendations. On one side, the World Health Organization (WHO) did not recommend using face masks as a preventive measure [1], in the absence of extensive scientific evidence on the matter. Till May 2020, there were no high quality controlled trials addressing the question of wearing masks by the general population as a protective measure to contain COVID-19. In the absence of evidence related explicitly to COVID-19, analogies were be made with similar types of viruses with high transmission rates, such as influenza or SARS. A recent meta-analysis looking into the effectiveness of the public’s use of non-pharmaceutical interventions in the transmission of influenza did not report supporting evidence for the use of face masks [2]. With the lack of strong evidence, the absence of supportive recommendations became more meaningful when considering the shortages of personal protective equipment, including face masks needed for health care workers worldwide.

Meanwhile, some public health agencies, such as the US Centers for Disease Control and Prevention (CDC), initially aligned with WHO’s recommendations, has later shifted to recommend the use of “*cloth face coverings”* [3] as a way to reduce the spreading of the virus. The European Centers for Disease Control and Prevention (ECDC) adopted a more cautious approach while highlighting the caveats of improper use and stating that wearing non-medical masks may be considered in situations where proper physical distancing cannot be maintained [4]. The rationale for this approach may be based on various assumptions: some recent evidence showed that COVID-19 is transmitted by asymptomatic people [5]; there was some evidence suggesting that people wearing face masks were less likely to transmit influenza compared to people not wearing face masks [6]; and mathematical modeling that pointed to around 20% decrease in transmission of influenza with the use of face masks by the public [7]. Another argument in support of the use of masks is the “precautionary principle” suggested in a recent systematic review [8], where Greenhalgh and colleagues argued that the benefits of using masks outweigh the risks of creating potential harm.

Amidst these opposing views and despite the lack of clear guidance and for rational use of face masks [9], countries have adopted a variety of recommendations, some following the mandatory face masks policies adopted in Asian countries [9]. However, little is known about how state decisions are made and followed in settings with weak health and public policy systems, such as Lebanon or similar countries in the Middle East and North Africa region. In fact, the notion and type of benefits o harm from mass use of face masks vary in each context and warrants assessment based on the prevailing conditions and social norms in a given population.

Lebanon still follows many laws inherited from the Ottoman Empire and the French mandate, and current policies are based on common law, instead of evidence. In the absence of clear national social and health policies and unified health promotion strategies, the private sector provides out of pocket curative care. The state constitution has allowed the personal status codes to be ruled by sectarian courts, while prominent state positions continue to be allocated to persons affiliated to sects instead of being appointed by merit or competence. Inherent economic post-civil war decline, social inequalities, mismanagement of national resources, and environmental degradation plague the country. These factors have created a chronic mistrust in the state and its ruling elite. In response to these dire conditions, a national revolution started in October 2019. The COVID-19 pandemic hit the country on February 21, 2020, and the newly formed government scrambled to put together an action plan in response, succeeding in flattening the curve but with huge economic costs borne by the population. The use of face masks has been extensively enforced throughout this outbreak. Despite the imposed public health measures of physical distancing, quarantine, and lockdown, the state health authority seems to have adopted a biomedical approach in this matter, rather than a systemic, holistic public health perspective. In this highly medicalized society, the mandatory use of face masks is another example of how clinical settings are extrapolated to the community and how global trends are adopted without critically considering the local context, implementation challenges, and long-term consequences.

The enforcement of such regulation has resulted in reality with potentially adverse impacts on the course of the outbreak. Using masks without proper instructions has given people a false sense of security, leading them to disregard physical distancing. According to the WHO’s guidance, this is one of the major factors that policymakers should consider before recommending the use of face masks [1]. Self-contamination is highly probable, as people are seen repeatedly touching their faces while wearing masks, positioning them below their mouths when speaking and removing them often and improperly. Improper use of face masks is also seen among government officials when appearing on the local media and reporting about the outbreak. Disposable masks are also littering streets, exacerbating the environmental health problems amidst the lack of a waste management strategy.

In a country lagging in evidence-based health policies, the risks and benefits of wearing masks are not weighed when making such recommendations. Yet, face masks are implicitly enforced in some public spaces. It was ironic to witness security forces mandating the use of face masks for people driving alone in a car, knowing well that seatbelt use is not enforced, despite a long-standing road safety law, which includes seat belt use. In a collectivist culture such as Lebanon, there is a need for contextualized, tailored communication on the notion of physical distancing in order to minimize the implementation challenges of the use of face masks by the public and the false sense of security they foster. Considering the impact of this pandemic, assumptions of a low level of harm [9] cannot apply universally, and there is a need to evaluate the impact of mass use of face masks in the Lebanese context.

**Figure:**
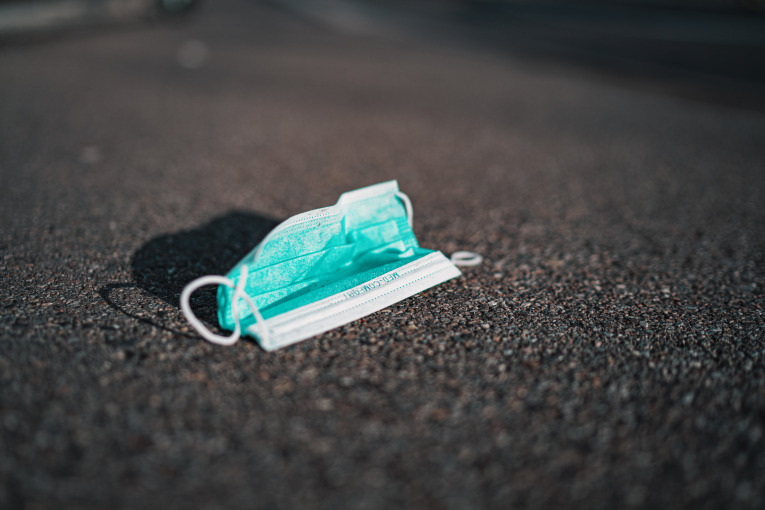
Photo: From https://www.barfuss.it/file/claudio-schwarz-purzlbaum-zh-btvpbcdw-unsplashjpg.

Currently, governments embarking in easing the restrictive measures of the lockdown are considering recommendations about the use of face masks by the general public. There is an urgent need for health authorities to follow effective risk communication guidelines [10] and provide the rationale and clear instructions for using face masks, while reinforcing the primary messages of physical distancing and hand hygiene [1]. In the absence of such efforts, we might witness a situation where face masks will result in more harm than good for the general public.
